# Gut microbiota dynamics in carnivorous European seabass (*Dicentrarchus labrax*) fed plant-based diets

**DOI:** 10.1038/s41598-020-80138-y

**Published:** 2021-01-11

**Authors:** Cláudia R. Serra, Aires Oliva-Teles, Paula Enes, Fernando Tavares

**Affiliations:** 1 CIMAR/CIIMAR - Centro Interdisciplinar de Investigação Marinha e Ambiental, Terminal de Cruzeiros do Porto de Leixões, Universidade do Porto, Av. General Norton de Matos s/n, 4450-208 Matosinhos, Portugal; 2 Departamento de Biologia, Faculdade de Ciências, Universidade do Porto, Rua do Campo Alegre S/N, Ed. FC4, 4169-007 Porto, Portugal; 3 CIBIO - Centro de Investigação em Biodiversidade e Recursos Genéticos, InBIO - Laboratório Associado, Universidade do Porto, Campus Agrário de Vairão, 4485-661 Vairão, Portugal

**Keywords:** Microbiology, Microbial communities

## Abstract

A healthy gastrointestinal microbiota is essential for host fitness, and strongly modulated by host diet. In aquaculture, a current challenge is to feed carnivorous fish with plant-feedstuffs in substitution of fish meal, an unsustainable commodity. Plants have a limited nutritive value due to the presence of non-starch polysaccharides (NSP) which are not metabolized by fish. In this work we assessed the effects of NSP-enriched diets on European seabass gut microbiota and evaluate the selective pressure of plant feedstuffs towards gut microbes with NSP-hydrolytic potential, i.e. capable to convert indigestible dietary constituents in fish metabolites. Triplicate groups of European seabass juveniles were fed a fish meal-based diet (control) or three plant-based diets (SBM, soybean meal; RSM, rapeseed meal; SFM, sunflower meal) for 6 weeks, before recovering intestinal samples for microbiota analysis, using the Illumina’s MiSeq platform. Plant-based diets impacted differently digesta and mucosal microbiota. A decrease (*p* = 0.020) on species richness, accompanied by a decline on the relative abundance of specific phyla such as Acidobacteria (*p* = 0.030), was observed in digesta samples of SBM and RSM experimental fish, but no effects were seen in mucosa-associated microbiota. Plant-based diets favored the Firmicutes (*p* = 0.01), in particular the Bacillaceae (*p* = 0.017) and Clostridiaceae (*p* = 0.007), two bacterial families known to harbor carbohydrate active enzymes and thus putatively more prone to grow in high NSP environments. Overall, bacterial gut communities of European seabass respond to plant-feedstuffs with adjustments in the presence of transient microorganisms (allochthonous) with carbohydrolytic potential, while maintaining a balanced core (autochthonous) microbiota.

## Introduction

The gastrointestinal tract is one of the most crowded bacterial communities on earth. Gut microbe's existence and influence on host physiology has been acknowledged for decades^1–4^. In healthy conditions, a mutually beneficial relationship is established between the host and its gut microbiota: the host provides a favorable niche for bacterial growth, with stable nutrient supply, while gut-bacteria perform or facilitate a series of digestive, metabolic, and immune-stimulating processes vital for host fitness^5^. Disturbances on this equilibrium leading to an imbalanced gut microbiota, also called dysbiosis, are linked to the development of multifactorial diseases in humans^6–8^, but also in farm animals^9,10^, including cattle^11–15^, swine^16,17^, poultry^18,19^, and farmed fish^20,21^. Diet has a tremendous influence on gut-microbiota composition and equilibrium^22–26^. This is particularly important in animal nutrition and production, where industry trends dictate a continuous evolution of raw materials, feedstuffs, and supplements used to feed farmed animals^27,28^.

Such tendencies are also verified in aquaculture, with the further attempt to feed carnivorous fish with plant-feedstuffs^27,29,30^. Traditionally, aquaculture production of carnivorous fish relies on fishmeal, which is an excellent protein source^31^, but also an unsustainable commodity, mainly provided by fisheries, whose availability for a rapidly growing aquaculture is decreasing. Plant feedstuffs, with world-wide production and attractive prices, are considered sustainable alternatives to fishmeal^30^. Despite their high availability, plant feedstuffs nutritive value for carnivorous fish is limited by the presence of anti-nutritional factors, including high levels of non-starch polysaccharides^30,32–34^.

As for other animal species, also in fish, ecology and diet are strongly correlated with digestive capacity^20,35–37^. While herbivorous fish possess longer intestines and strong carbohydrolytic capacity, carnivorous fish digestive systems are shorter and more proteolytic. Fish do not possess the necessary carbohydrate-active enzymes to hydrolyze non-starch polysaccharides^38^, that remain indigestible, interacting with fish gut epithelium and gut- microbiota, contributing to fish physiological and inflammatory imbalances^20,39^.

Recently, we were able to isolate several bacterial isolates, two of them patented (PCT/IB2019/059131), with a broad and potent carbohydrolytic activity from the gut of European seabass (*Dicentrarchus labrax)*, a carnivorous marine fish species, fed with plant-based diets^40^. In that work, we hypothesized that the plant-based diets used acted as a selective pressure to modulate the fish gut microbiota towards enrichment of bacteria capable of digesting those non-starch polysaccharides. To confirm that hypothesis, here we analyze, through 16S rRNA amplicon sequencing, the dynamics of gut microbiota of European seabass juveniles fed the same challenging plant-based diets to elucidate putative selective pressures favoring a gut microbiota more fit to metabolize non-starch polysaccharides. This knowledge might contribute to identify new probiotics and improve aquaculture practices of carnivorous fish fed with plant-based diets.

## Results

### The European seabass mucosa-associated gut microbiota is more stable than the digesta-associated microbiota

The dietary inclusion of SBM, SFM, or RSM had no effect on European seabass growth performance, feed intake, feed efficiency, protein efficiency ratio and N intake (Table 1). Digesta and mucosa gut microbiota assessed by 16S rRNA amplicon sequencing provided at least 190 000 read counts per sample. After pre-processing, a total of 427 284 high-quality reads were clustered into 2849 OTUs at 97% identity threshold (Tables S1 and S2).

**Table 1:** Growth performance and feed utilization efficiency of European sea bass fed the experimental diets.

Diets^1^	CTR	SBM	RSM	SFM
Final body weight (g)	73.4 ± 5.2	70.0 ± 2.6	73.9 ± 4.2	71.4 ± 0.9
Daily growth index^2^	2.07 ± 0.22	1.93 ± 0.11	2.10 ± 0.18	1.99 ± 0.04
Feed intake^3^ (g kg^−1^ABW day^−1^)	17.7 ± 1.4	19.8 ± 1.5	18.4 ± 1.6	20.7 ± 0.3
Feed efficiency^4^	1.01 ± 0.11	0.85 ± 0.06	0.93 ± 0.16	0.82 ± 0.05
Protein efficiency ratio^5^	2.15 ± 0.24	1.82 ± 0.13	2.02 ± 0.35	1.78 ± 0.12
N Intake^3^ (g kg^−1^ABW day^−1^)	1.22 ± 0.09	1.35 ± 0.10	1.25 ± 0.11	1.41 ± 0.02

Values presented as means ± standard deviation (± SD) (n = 3 per treatment pooled from 6 fish).

^1^CTR, control fishmeal based diet; SBM, soybean meal based diet; RSM, rapeseed meal based diet; SFM, sunflower meal based diet.

^2^DGI: ([final body weight^1*/*3^ − initial body weight^1*/*3^]/time in days) × 100.

^3^ABW: average body weight (initial body weight + final body weight)/2.

^4^Feed efficiency (FE) = (wet weight gain/dry feed intake).

^5^PER: (wet weight gain/crude protein intake).

Contaminant sequences of chloroplasts, common in NGS studies, in particular in those analyzing herbivores guts or plants-associated microbiota, due to their 16S high homology to that of bacteria, were removed from the downstream analysis, as previously reported in similar studies^41–43^.

**Figure 1:**
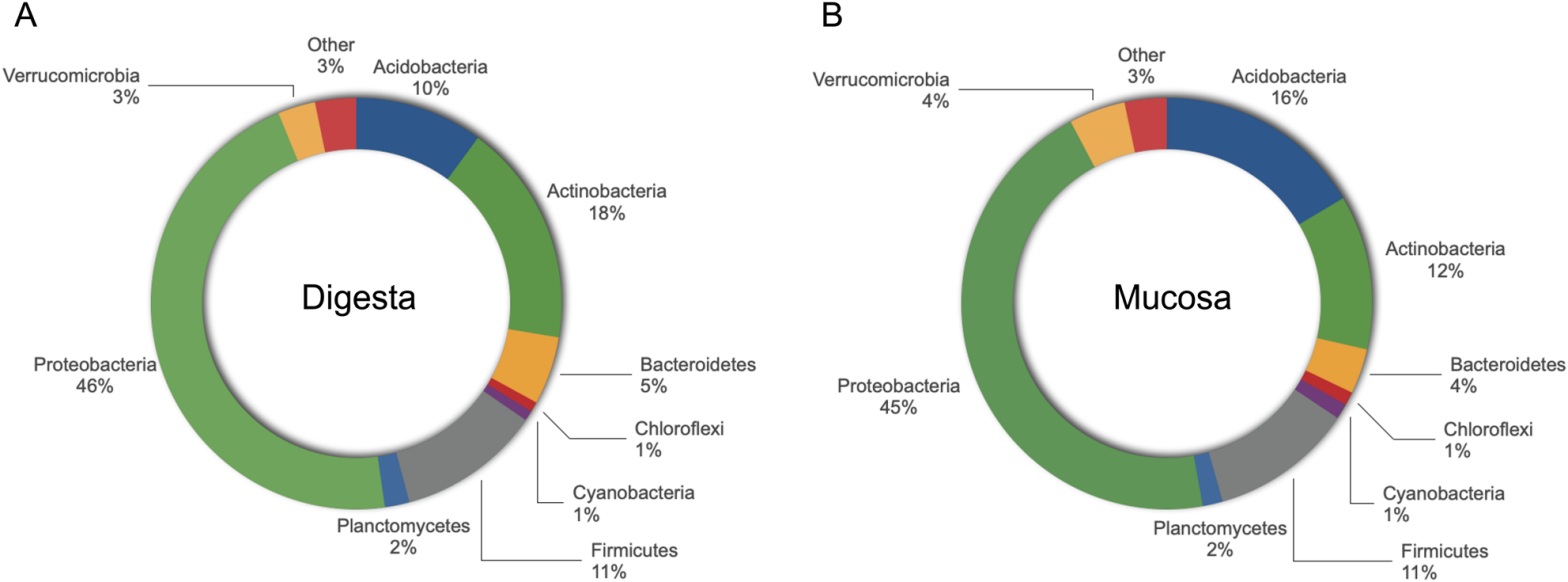
Bacterial phyla diversity obtained from digesta (**A**) and mucosa (**B**) samples of European sea bass fed the experimental diets for 45 days, after NGS analysis by Illumina MiSeq. There are no significant differences between both intestinal compartments, although the Acidobacteria are more abundant at the mucosal level while the Actinobacteria show higher values at the luminal level.

Taxa showing a mean proportion of 1% or higher in any experimental feeding condition (CTR, SBM, RSM & SFM) or intestinal sample (Digesta & Mucosa) were considered as the most abundant. Proteobacteria was the predominant phylum, accounting for more than 45% of the sequencing reads in both digesta and mucosa samples (Fig. 1). The Firmicutes were equally represented in both digesta and mucosa samples. On the contrary, Acidobacteria and Actinobacteria phyla showed a 6% difference in their representation, with Actinobacteria being more abundant in digesta and Acidobacteria in mucosa samples (Fig. 1). Other phyla, including Cyanobacteria, Bacteroidetes, Verrucomicrobia, Planctomycetes and Chloroflexi were less represented in both digesta and mucosa samples (below 5% each). Regarding individual OTUs, digesta and mucosa samples shared 520, while 378 OTUs were digesta-specific and 57 OTUs were mucosa-specific (Table S2 and Table S3). These later included organisms from *Brevimena*, *Gardnerella*, *Nakamurella*, *Pasteurella, Emticicia, Schlesneria, Kingella, Azotobacter, Solitalea, Alkanibacter*, *Anaerospora, Megasphaera* and *Candidatus_Entotheonella* genera.

**Figure 2:**
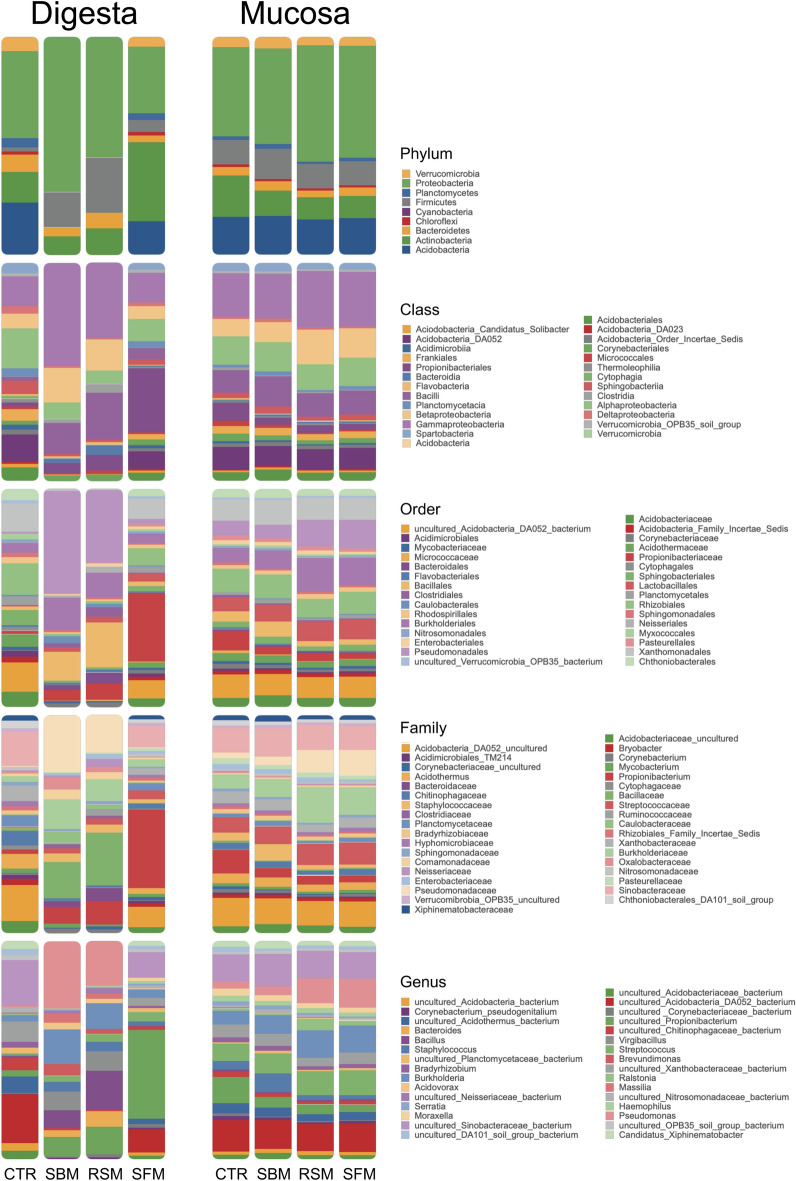
Relative bacterial abundance (*y*-axis) at Phylum, Class, Order, Family and Genus Taxonomic levels (from top to bottom), in Digesta and Mucosa samples of European sea bass feed the experimental diets for 45 days (*x*-axis): CTR, control fishmeal based diet; SBM, soybean meal based diet; RSM, rapeseed meal based diet; SFM, sunflower meal based diet. Presented are taxa with a mean proportion ≥ 1% in any experimental feeding condition.

The variations on microbial richness, diversity, and evenness indices, obtained from the NGS data, are presented in Table 2. Dietary replacement of FM by SBM or RSM decreased (*p* = 0.020) the *Chao1* species richness estimator index of the digesta-associated microbiota. The other diversity indices did not significantly differ between the experimental diets, but a tendency to their decrease was visible in the digesta samples from plant-based diets when compared to the FM-based control diet. On the contrary, mucosa-associated microbiota was stable, with its diversity indices remaining unaffected upon the inclusion of plant-feedstuffs on European seabass diets (Table 2). Such higher stability of mucosal microbiota relative to digesta-associated microbiota was observable independently of the taxonomic level analyzed (Fig. 2). For instance, the relative abundance of the different phyla in response to the dietary incorporation of RSM, SBM or SFM, despite observable variations (e.g. RSM & SBM diets favor the Firmicutes, while the dietary incorporation of SFM raised the Actinobacteria levels) was more stable in mucosal microbiota than in the luminal (digesta) microbiota, which seems to be more variable and diet-dependent. Such a trend is maintained at the other taxonomic levels analyzed (Class, Order, Family, and Genus). Regardless of their location (intestinal lumen or intestinal mucosa), Alphaproteobacteria, Betaproteobacteria, Gammaproteobacteria, Bacilli, and Actinobacteria were the predominant Classes (Fig. 2). Also, the Acidobacteria_DA052 was present at high and constant levels in all samples tested, with the exception of digesta from SBM and RSM diets. The most abundant Orders and Families fall within the previously mentioned most abundant Classes, namely: (1) Rhizobiales_Xanthobacteraceae (Alphaproteobacteria); (2) Burkholderiales_Burkholderiaceae (Betaproteobacteria); (3) Pseudomonadales_Pseudomonadaceae and Xanthomonadales_Sinobacteraceae, both Gammaproteobacteria; (4) the Bacilli Bacillales_Bacillaceae (in digesta SBM and RSM samples), Bacillales_Staphylococcaceae and Lactobacillales_Streptococcaceae. It is also worth noticing the high amount of Propionobacteriales_Propionibacteriaceae (Actinobacteria) found in digesta samples from fish fed the SFM diet. The predominant genera were mainly uncultured bacteria from the Families identified above, but *Burkholderia*, *Pseudomonas, Staphylococcus and Streptococcus* could be found at high levels in all samples (Fig. 2; Table S4). Additionally, *Bacillus* and *Virgibacillus* were the identified predominant genera among digesta samples of fish fed the SBM and RSM diets, while in SFM an uncultured Propionibacterium dominated. As observed in the superior taxonomic levels, an uncultured genus from Class Acidobacteria_DA052, was present at high and constant levels in all samples tested, with the exception of digesta from SBM and RSM diets.

**Table 2:** Ecological parameters obtained from NGS analysis of the intestinal and mucosal microbiota recovered from European sea bass at 45 days after feeding the experimental diets (CTR, fishmeal based diet; SBM, soybean meal based diet; RSM, rapeseed meal based diet; SFM, sunflower meal based diet).

Diets^1^	CTR	SBM	RSM	SFM
**DIGESTA**				
Richness^2^	836 ± 14^b^	333 ± 13^a^	317 ± 3^a^	667 ± 214^ab^
Diversity^3^	8.6 ± 0.04	4.4 ± 0.4	5.4 ± 0.2	6.5 ± 2.8
Evenness^4^	1 ± 0.0003	0.8 ± 0.02	0.9 ± 0.02	0.9 ± 0.2
**MUCOSA**				
Richness^2^	433 ± 143	556 ± 26	490 ± 21	573 ± 25
Diversity^3^	7 ± 0.2	7.3 ± 0.1	6.8 ± 0.1	7.1 ± 0.6
Evenness^4^	1 ± 0	1 ± 0	1 ± 0	1 ± 0

Values presented as means ± standard deviation (± SD) (n = 3 per treatment pooled from 6 fish).

One-way ANOVA: * *p* < 0.05. Different letters stand for significant differences between diets.

^1^CTR, control fishmeal based diet; SBM, soybean meal based diet; RSM, rapeseed meal based diet; SFM, sunflower meal based diet.

^2^*Chao1* species richness: S_*Chao1*_ = S_obs_ + n_1_^2^/2n_2_, where S_obs_ is nr of species, n_1_ singletons , and n_2_ doubletons.

^3^Shannon’s diversity index: H’ = − ∑(Pi(lnPi)), whereas *Pi* is the nr of individuals of the ith species.

^4^Simpson’s Evenness Index: E = (1/∑Pi2)/S, where S is ty number of species.

### Plant-based diets favor plant-associated bacterial taxa

**Figure 3:**
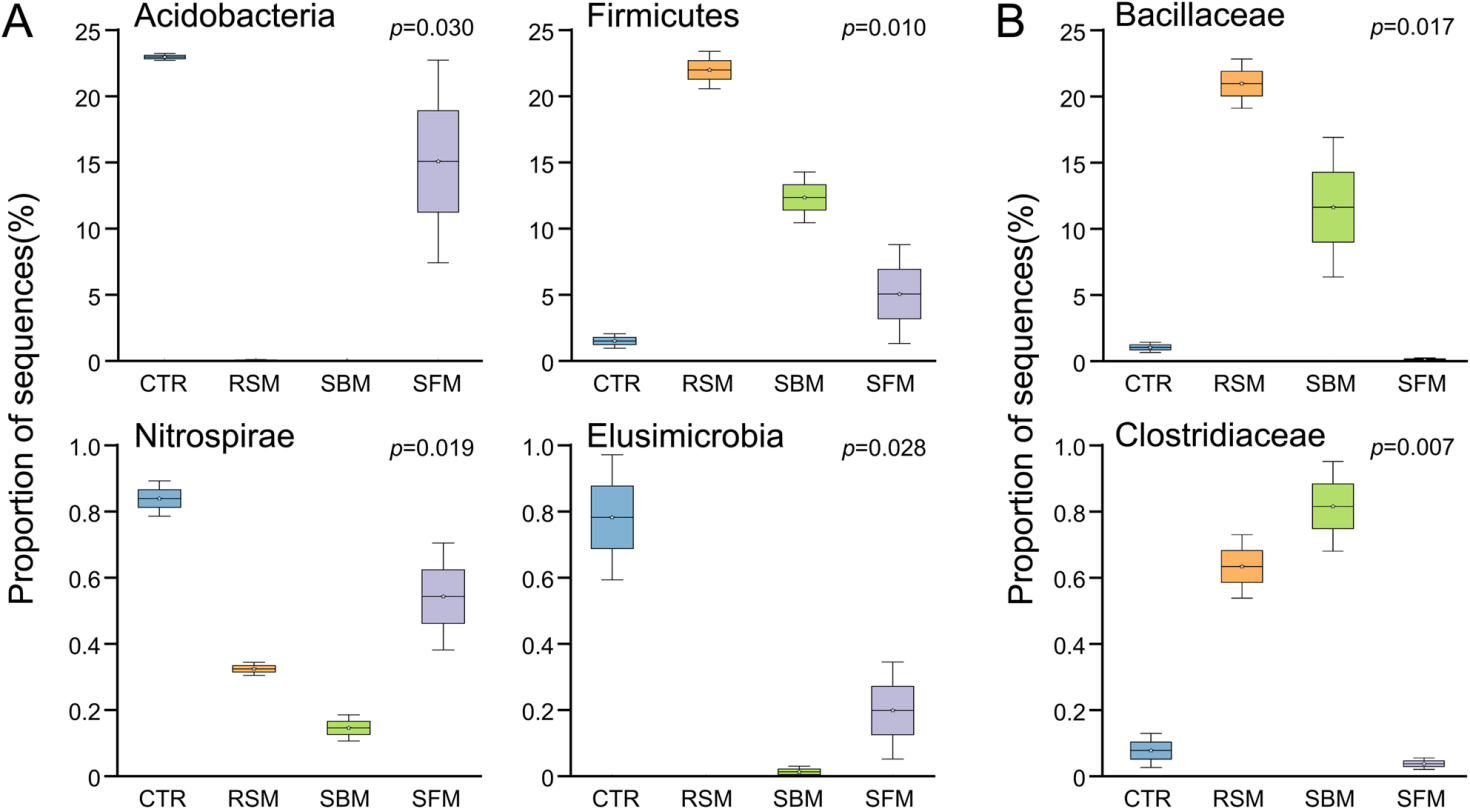
Relative proportion of sequences (*y*-axis) derived from the NGS data, in digesta samples of European sea bass fed the experimental diets for 45 days (*x*-axis): CTR, control fishmeal based diet; SBM, soybean meal based diet; RSM, rapeseed meal based diet; SFM, sunflower meal. Incorporation of SBM and RSM diminishes the *Acidobacteria* (*p* = 0.030), *Elusimicrobia* (*p* = 0.028) and *Nitrospirae* (*p* = 0.010) phyla. On contrary, SBM and RSM PF-based diets favor the *Firmicutes* (*p* = 0.01), in particular the Bacillaceae (*p* = 0.017) and Clostridiaceae (*p* = 0.007).

The statistical analysis of the mean relative frequency within each taxonomic level in both mucosal and digesta samples is presented in Supplementary Tables S5—Digesta, and S6—Mucosa. Dietary incorporation of plant ingredients (SBM, RSM or SFM) significantly affected (increased or decreased) the abundance of 5 Phyla, 23 Classes, 34 Orders, 53 Families, and 74 Genera at digesta level. In mucosal samples, the number of affected taxa was smaller (0 Phyla, 3 Classes, 4 Orders, 7 Families, and 11 Genera). Regarding taxa with a mean proportion of 1% or higher (represented in bold in Supplementary Tables S5 and S6), while in Mucosa there was only 1 Family (Pseudomonadaceae) and 1 Genus (*Ralstonia*) affected by the experimental feeding conditions, both increasing with dietary incorporation of plant ingredients (SBM, RSM or SFM) relative to the CTR diet (Table S5), in Digesta, 40 taxa (3 Phyla, 6 Classes, 10 Orders, 11 Families, and 10 Genera) significantly differed between experimental groups. A decrease in phyla Acidobateria (*p* = 0.030), Nitrospirae (*p* = 0.019), Elusimicrobia (*p* = 0.028), and Chlorofloxi (*p* = 0.007), and an increase (*p* = 0.010) of Firmicutes was observed when any of the plant ingredients were incorporated in the diet (Fig. 3A; Table S5).

Within the Firmicutes phylum, diets SBM and RSM favored in particular the Bacillaceae (*p* = 0.017) and Clostridiaceae (*p* = 0.007) (Fig. 3B; Table S5). Other families whose representation was increased upon plant-feedstuffs incorporation in the diets included the Bifidobacteriaceae (*p* = 0.005) (Phylum Actinobacteria); the Alteromonadaceae (*p* = 0.001), Pseudomonadaceae (*p* = 0.008), and Rhodocyclaceae (*p* = 0.007), all Proteobacteria (Table S5); and the Flavobaceriaceae (*p* = 0.036) (Phylum Bacteroidetes).

**Figure 4:**
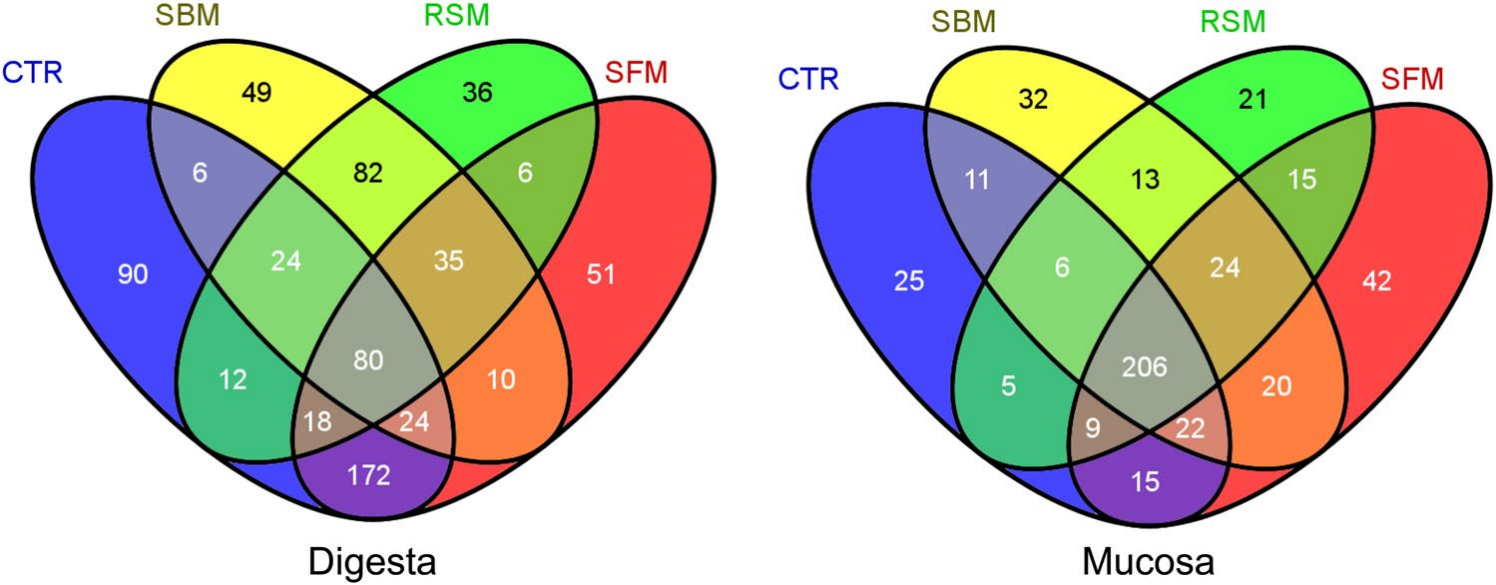
Venn diagram representation of shared and unique genera across the experimental feeding groups CTR (control fishmeal based diet), SBM (soybean meal based diet), RSM (rapeseed meal based diet) and SFM (sunflower meal, based diet), in Digesta and Mucosa samples, using Venny (https://bioinfogp.cnb.csic.es/tools/venny_old/venny.php).

Regardless of the diets provided, a core microbiota could be identified in both digesta and mucosa samples (Fig. 4; Table S7). While in Mucosa samples the great majority of genera (206) were present in all diets, in Digesta samples different genera could be assigned to samples of different experimental diets, confirming the higher stability of mucosal microbiota versus digesta microbiota.

## Discussion

Fish digestive system anatomy and functioning are adapted to feeding habits^35^, with omnivorous and herbivorous fish having longer digestive tract and higher carbohydrolytic enzyme activity than carnivorous fish, thus being more adapted to deal with plant feedstuffs. However, current industry trends dictate feeding carnivorous fish with plant-based diets. This practice not only impacts on fish gastrointestinal health, since plants carry antinutritional factors that might impair carnivorous fish digestive function^33,34^, but also strongly modulate fish gut microbiota^20,35,44,45^. Because gut microbiota composition is diet-dependent and influences host health and well-being, assessing what impact feeding carnivorous fish with plant-based diets has on fish gut microbiota is essential to fully evaluate current aquaculture feeding strategies. This theme has been recently addressed, through high-throughput sequencing, in a few carnivorous aquaculture fish species, mainly in the salmonids Atlantic salmon (*Salmo salar*), brown trout (*Salmo trutta*), or rainbow trout (*Oncorhynchus mykiss*)^41,46–49^, but also in other teleosts such as gilthead seabream (*Sparus aurata*)^50–52^, Senegalese sole (*Solea senegalensis*)^53^ or sablefish (*Anoplopoma fimbria*)^54^. Regarding our target-species, the European seabass, its gut microbiota has been characterized through high-throughput sequencing in a few studies, focused on the development of fish tracing tools^55^, on fish geographical location^56^, on fish feeding with functional diets containing immunostimulants (B-glucans^57^, poly-β-hydroxybutyrate^58^), different salt concentrations^59^, and unbalanced diets^60^. No high-throughput study done on European seabass has so far addressed the impact of plant-based diets on gut microbiota. The only culture-independent approach to such characterization has been through Denaturing Gradient Gel Electrophoresis or DGGE^61^ that, although sufficient to clarify differences in bacterial community gross composition, fails in characterizing phylogenetic diversity in detail^62^.

In the present study, we describe how plant-based diets modulate both digesta and mucosal microbiota of European seabass. The data showed that plant-based diets, namely SBM, RSM, and SFM, impact differently digesta and mucosa microbiota. While a decrease of Shannon’s diversity index characterized by a lower microbial richness and a decrease of microbial diversity was observed in digesta samples, mucosal microbiota was shown to be less-diet dependent and disclose a larger diet-independent core microbiota. In mucosa, 44% (206 out of 466) of the identified genera were present in all fish samples, independently of the feeding group, while only 11% (80 out of 695) genera were common to all diets in digesta samples. A core microbiota less sensible to dietary changes has been reported previously in other carnivorous species, namely rainbow trout^63^ and Atlantic salmon^64^, but those studies did not separately analyze mucosal and digesta samples of the same fish. On the contrary, Gajardo et al.^43^ made a distinction between mucosal and digesta compartments, describing a richer and more diverse digesta-associated microbiota in Atlantic salmon, but without using different diets (all fish were fed the same commercial diet fulfilling the species requirements). Similarly, to Atlantic salmon, we also observed a higher total number of OTUs in digesta samples, and of digesta-specific OTUs indicating that in European seabass, under our experimental conditions, only a fraction of digesta-associated bacteria is capable of effectively colonize the fish gut, by associating with its mucosa. Nevertheless, such fraction is not negligible, since from 967 OTUs, 529 were shared between digesta and mucosa and 57 were mucosa-specific OTUs. The observation that European seabass mucosa-associated gut microbiota is more stable (less diet-dependent) than the digesta-associated one, suggests that bacterial gut communities of European seabass respond to dietary changes by maintaining a balanced core (autochthonous microbiota), with adjustments in the presence of transient microorganisms (allochthonous microbiota). Taking this observation in consideration, to further understand European sea bass response to the incorporation of plant-feedstuffs, we focused our analysis in the dynamics of digesta-associated microbiota. Nevertheless, as recently highlighted by Berg et al*.*^65^ “*defining the core microbiota facilitates discrimination of the stable and permanent members of a microbiome from populations that may be intermittent, associated only with specific microbiome states, or restricted to specific environmental conditions*”. Also, although the importance of rare taxa to host and microbiota functions is increasingly recognized, the identification of a common core microbiota (highly prevalent taxa found across the majority of hosts within a population) might reveal key members of the gut community with particular relevance to host biological functions and fitness^66^. A core microbial community composed of 7 bacterial genera persistant across different habitats, diets, gut parts, and importantly, across different fish species including the carnivorous European seabass, salmon, trout, three-spined stickleback and perch, the herbivorous tilapia and the omnivorous zebrafish was recently described^67^. Five of those genera (Pseudomonas, Acinetobacter, Stenotrophomonas, Aeromonadaceae genus and Comamonadaceae genus) were also found as part of the European seabass core microbiota identified in our study, while one (Janthinobacterium) was only detected in the digesta of fish fed the CTR diet and another (Morganella) was not detected in any sample.

The overall microbial composition of the European seabass gut was similar to that recently described in other teleosts^20,41–43^ and in particular in other studies on European seabass^56,60^. Gut microbiota was dominated by Proteobacteria, Firmicutes, Acidobacteria, and Actinobacteria and their relative abundances were not significantly different between digesta and mucosa intestinal samples. Recently a comprehensive assessment of over 200 bacterial isolates has shown that bacteria with a broad and potent carbohydrolytic activity are present in the gut of European seabass fed plant-based diets^40^, suggesting that plant-based diets could act as a selective pressure to modulate the carnivorous fish gut microbiota towards an enrichment of carbohydrolytic bacteria. While this former study was culture-based and focused on sporeformers, therefore unable to disclose all bacterial diversity, the current work provides new insights about the total bacterial diversity and predominant bacterial genera selectively promoted by the diets used, unbiased by culturability. Carnivorous fish were reported to have less diverse microbiota^68^ than herbivorous fish and dominant genera were shown to be different between carnivorous and herbivorous fish^35,69^. Some authors suggested that increasing herbivory in fish could lead to gut microbiota diversification, as seen in mammals^68^, but under our experimental conditions, feeding carnivorous European seabass with plant-based diets resulted in a decrease in gut bacterial richness (at digesta level), accompanied by a decline on the relative abundance of specific phyla such as Acidobacteria, Elusimicrobia, and Nitrospirae. Because contradictory effects (both null, positive and negative) of fish-meal substitution by plant-feedstuffs on gut microbiota richness and diversity have been previously reported in other aquaculture carnivorous fish species, including gilthead seabream, rainbow trout or Atlantic salmon^47,48,52,63^, any interpretation of such observation would be merely speculative. Interestingly, only Firmicutes increased in digesta samples of European seabass-fed plant-based diets. The Firmicutes are a phylum of highly diverse and widespread organisms with more than 250 genera, whose presence in animals gut in general, and in the fish gut in particular, has been extensively acknowledged^5,20,21^. In humans and mammals’ gut, Firmicutes abundance and the relation to Bacteroidetes numbers (where both represent 90% of the total gut microbiota) has been used as a measure of microbiota balance. Briefly, it has been suggested that the lower the Firmicutes: Bacteroidetes ratio is, the healthier is the gut^5^. In fish, Bacteroidetes are not as relevant as in mammals, but instead, Proteobacteria are repeatedly described as the most abundant Phylum in microbiota characterization studies^20,21,70^. A recent study done in rainbow trout, found out Firmicutes and Proteobacteria to be “*particularly discriminatory for diet type*”, with plant-based diets favoring a higher Firmicutes: Proteobacteria ratio than animal-based diets^42^. Although no significant changes in Proteobacteria abundance were observed in the current work, the fact that Firmicutes increased in gut digesta of fish challenged with any of the plant-based diets tested (SBM, RSM, and SFM) raised the Firmicutes: Proteobacteria ratio. Within the Firmicutes, plant-based diets favored, in particular, the Bacillaceae and Clostridiaceae, two bacterial families that are known to harbor carbohydrate-active enzymes, and thus putatively more prone to grow in high fiber environments^71,72^. Species of both families have the particularity of producing endospores that assure the survival of the species under potentially fatal insults (e.g. radiation, desiccation, high pressure, high temperatures)^73^. Bacillaceae are mostly aerobic or microaerophilic organisms, while Clostridiaceae are mainly anaerobic.

In the present work, sporulating Firmicutes are mainly represented by the genus *Bacillus* together with *Virgibacillus* whatever the digesta sample. These data are aligned with the culture-based assessment of aerobic endosporeformers to isolate carbohydrolytic bacteria from European seabass gut fed with plant-based diets^40^. Regarding highly represented genera from other phyla, besides *Pseudomonas* (Gammaproteobacteria), *Burkholderia* (Betaproteobacteria), uncultured organisms from Gammaproteobacteria, Acidobacteria and Actinobacteria (assigned to the genus *Propionibacterium),* were the predominant genera among digesta samples of SBM and RSM. This later genus was also the most abundant in SFM digesta samples. However, within those highly abundant genera, only *Pseudomonas* (a Proteobacteria) significantly increased in the gut of plant-fed fish, with exception of those fed SFM. *Pseudomonas* genus contains well-known pathogenic species, such as *P. aeruginosa*, but also potential probiotics for the aquaculture industry, being abundant in aquatic environments and in fish gut, including that of European seabass^20,43,53,56,60^. Some *Pseudomonas* spp. have been described to have carbohydrolytic activity^74^, and were reported to also increase in gilthead seabream fed plant-based diets^52^.

Genera within the Bacillaceae and Clostridiaceae significantly affected by plant-based diets belonged to less representative OTUs, namely *Oceanobacillus, Pausicalibacillus,* and *Lentibacillus* from the first family, and *Clostridium* from the latest. *Oceanibacillus*, *Pausicalibacillus,* and *Lentibacillus* are all halophilic Bacillaceae, commonly found in high salt ecosystems such as salterns, whose carbohydrolytic activity has been poorly characterized^75–78^. In agreement, in our previous work, the best carbohydrolytic strains belonged to the *Bacillus* genus, and not to less abundant genera such as *Oceanobacillus*^40^. On the contrary, *Clostridium* spp. carbohydrolytic capacity has been acknowledged previously^74,79,80^. *Clostridium* is a genus with problematic pathogenic species both for humans and animals, such as *C. difficile*, *C. botulinum,* and *C. perfringens*, and although no Clostridial disease has been described in fish, their presence in the fish gut, both in freshwater and marine species, is repeatedly reported^20,69,81,82^. Estruch et al*.*^52^*,* observed their presence in gilthead seabream fed plant-based diets but not fish-meal based-diets, and in European seabass, their numbers increased in fish fed a low-fish meal/high non-starch-polysaccharide diets^61^. Clements et al*.*^81^ and Liu et al*.*^69^ even reported Clostridia to dominate the gut of herbivorous marine and fresh-water fish species, respectively. Although *Clostridium* was not one of the prevalent genera in our study, its significant increase upon plants incorporation into European seabass diets might indicate that they play a key role in helping carnivorous fish to tolerate plant feedstuffs. A similar trend was seen in salmon^41^, where increasing dietary carbohydrates mostly affected low-abundance bacteria, favoring those groups with carbohydrolytic potential. Altogether this study details how plant-based diets affect the gut microbiota of European seabass and elucidate the predominant bacterial taxa that might inform culture-based studies to isolate novel strains with carbohydrolytic potential. As the utilization of low-cost plant feedstuffs with high level of non-digestible carbohydrates including NSP, is a tendency in carnivorous fish aquafeeds production, the potential of such bacterial strains might be very important and deserves to be further exploited.

Plant-feedstuffs used in this study (SBM, RSM, and SFM) contain circa 22–24% of NSP components, most of which are pectic polysaccharides^83^. Galactose is the predominant sugar residue in SBM, arabinose in RSM, and xylose in SFM^83^. As recently described in zebrafish^84^, and extensively in mice and humans (reviewed in^85^), different polysaccharides, including different NSP, have different effects on gut microbiota. Some contribute to the maintenance of gut microbial homeostasis, while others potentiate gut dysbiosis. This microbiota modulation is dependent on the polysaccharide structure, its fermentation by the gut bacteria and its direct interaction with the gut epithelium and mucus, which ultimately might result in physiological and inflammatory imbalances^83–85^. Although carbohydrates-metabolism has been exhaustively studied in different microorganisms, including gut ones, and there is enough genomic information (both from individual microorganisms and metagenomics studies) confirming that gut microorganisms possess the necessary enzymatic tools to metabolize different NSPs, it is not known which of these organisms are indeed capable of such metabolizing jobs within the complex context of natural gut communities and if metabolic pathways, capabilities and preferences determined in vitro will be replicated inside the gut. A sophisticated and targeted-approach was recently employed to reveal microbes within the mouse complex gut community with the capacity to utilize mucosal sugars^86^. Similar studies are needed to exploit specific NSP-microbiota interactions in aquaculture fish to fully unveil the underlying mechanisms determining the fate of specific NSP and its effect on fish performance, fish health and nutrient digestibility^39^.

In conclusion, feeding carnivorous fish species, such as European seabass, with plant-based diets, favors the presence of transient microorganisms with carbohydrolytic potential, without affecting the autochthonous microbiota. The question whether such microbiota modulation is temporary or could become permanent/established (at autochthonous level) if the dietary challenge would be prolonged enough, remains to be answered and is worth of investigating by long term studies.

## Materials and methods

All methods were carried out in accordance with relevant guidelines and regulations, namely in the construction of figures and their compliance with the digital image and integrity policies. All animal experiments were approved by the Animal Welfare Committee of the Interdisciplinary Centre of Marine and Environmental Research (CIIMAR) and carried out in a registered installation (N16091.UDER) and were performed by trained scientists (following FELASA category C recommendations) in full compliance with national rules and following the European Directive 2010/63/EU of the European Parliament and the European Union Council on the protection of animals used for scientific purposes.

### Diets composition

Four experimental diets (Table 3) were formulated, based on the ones we previously used^40^, to be isonitrogenous (47% crude protein) and isolipidic (17% crude lipid) and to contain 30% of soybean meal (SBM diet), 30% of rapeseed meal (RSM diet) or 30% of sunflower meal (SFM diet). A fish meal-based diet was used as the control diet (CTR diet). Fish oil and pregelatinized maize starch were used as the main lipid and carbohydrate sources, respectively. Bicalcium phosphate was added to adjust dietary phosphorus level. All diet ingredients were thoroughly mixed and dry-pelleted in a laboratory pellet mill (California Pellet Mill, CPM Crawfordsville, IN, USA), through a 3.0 mm die. Pellets were dried in an oven at 50 °C for 24 h, and then stored at − 20 °C until used. Ingredients and proximate composition of the experimental diets are presented in Table 3.

**Table 3:** Ingredients composition and proximate analysis of experimental diets.

Diets^a^	CTR	SBM	RSM	SFM
**Ingredients (% dry weight)**				
Fish meal^b^	60.2	38.7	45.2	48.1
Soy bean meal^c^	─	30.0	─	─
Rapeseed meal^d^	─	─	30.0	─
Sunflower meal^e^	─	─	─	30.0
Pregelatinized maize starch^f^	23.2	11.6	8.0	4.8
Fish oil	12.1	13.6	12.4	13.0
Bicalcium phosphate^g^	1.0	2.6	1.0	0.6
Choline chloride (50%)	0.5	0.5	0.5	0.5
Vitamin premix^h^	1.0	1.0	1.0	1.0
Mineral premix^i^	1.0	1.0	1.0	1.0
Binder^j^	1.0	1.0	1.0	1.0
**Proximate analysis (% dry weight)**				
Dry matter	91.5	92.4	92.7	93.5
Crude protein	46.9	46.5	46.3	46.4
Crude lipids	17.3	16.1	16.6	16.8
Ash	11.3	11.7	11.3	11.1

*DM* dry matter, *CP* crude protein, *CL* crude lipid.

^a^CTR, control fishmeal based diet; SBM, soybean meal based diet; RSM, rapeseed meal based diet; SFM, sunflower meal based diet.

^b^Steam Dried LT fish meal, Pesquera Diamante, Austral Group, S.A Perú (CP: 74.7% DM; GL: 9.8% DM).

^c^Sorgal, S.A. Ovar, Portugal (CP: 53.7% DM; GL: 2.1% DM).

^d^Sorgal, S.A. Ovar, Portugal (CP: 37.5% DM; GL: 4.0% DM).

^e^Sorgal, S.A. Ovar, Portugal (CP: 30.3% DM; GL: 1.0% DM).

^f^C-Gel Instant-12016, Cerestar, Mechelen, Belgium.

^g^Premix, Portugal (Calcium: 24%; Total phosphorus: 18%).

^h^Vitamins (mg kg^−1^ diet): retinol acetate, 18,000 (IU kg^−1^ diet); cholecalciferol, 2000 (IU kg^−1^ diet); alfa tocopherol acetate, 35; sodium menadione bisulphate, 10; thiamine-HCl, 15; riboflavin, 25; calcium pantothenate, 50; nicotinic acid, 200; pyridoxine HCl, 5; folic acid, 10; cyanocobalamin, 0.02; biotin, 1.5; ascorbic acid, 50; inositol, 400.

^i^Minerals (mg kg^−1^ diet): cobalt sulphate, 1.91; copper sulphate, 19.6; iron sulphate, 200; sodium fluoride, 2.21; potassium iodide, 078; magnesium oxide, 830; manganese oxide, 26; sodium selenite, 0.66; zinc oxide, 37.5; dibasic calcium phosphate, 8.02 (g kg^−1^ diet); potassium chloride, 1.15 (g kg^−1^ diet); sodium chloride, 0.44 (g kg^−1^ diet).

^j^Aquacube (guar gum, polymethyl carbamide, manioc starch blend, hydrate calcium sulphate) Agil, UK.

### Animals and experimental conditions

The experiment was performed following procedures previously described^40^, at CIIMAR, Porto University, Portugal, with European seabass (*Dicentrarchus labrax*) juveniles obtained from a commercial fish farm (Maresa S.A., Ayamonte, Huelva, Spain). After transportation to the experimental facilities fish were submitted to a quarantine period of 30 days and then transferred to the experimental system for adaptation to the experimental conditions for 15 days. Before the experimental period, fish were fed a commercial diet (48% protein, 11% lipids, 5% starch). The trial was performed in a recirculating water system equipped with 12 cylindrical fiberglass tanks of 100 l water capacity and thermo-regulated to 22.0 ± 1.0 °C. Tanks were supplied with a continuous flow of filtered seawater (2.5–3.5 l min^−1^) of 34.0 ± 1.0 g l^−1^ salinity and dissolved oxygen was kept near saturation (7 mg l^−1^). Thereafter, 20 European seabass with an initial mean body weight of 34.4 g were distributed to each tank and the experimental diets randomly assigned to triplicate groups. The trial lasted 45 days and fish were fed by hand, twice daily, 6 days a week, until apparent visual satiation. Utmost care was taken to avoid feed losses. The experiment was performed by accredited scientists (following FELASA category C recommendations) and was conducted according to the European Union directive 2010/63/EU on the protection of animals for scientific purposes.

### Sampling

Fish sampling was done essentially as previously described^40^. Briefly, fish in each tank were bulk weighed at the beginning and at the end of the trial, after one day of feed deprivation. For that purpose, fish were lightly anesthetized with 0.3 ml l^−1^ ethylene glycol monophenyl ether. After the final weighting, fish were fed for 3 more days, to minimize manipulation stress. Then, 3 fish per tank were randomly sacrificed 4 h after the first meal, to guarantee that intestines were full at sampling time, with an overdose of ethylene glycol monophenyl ether, for collection of biological samples under aseptic conditions.

To overcome inter-fish variation the resulting material was pooled into one sample per tank. Intestines (without pyloric caeca) were aseptically excised and digesta and intestinal mucosal tissue removed. Digesta was obtained by squeezing the entire intestine. Mucosa was obtained by scraping the internal intestinal mucosa after opening the intestines in their longitudinal axis. Both digesta and mucosa samples were immediately frozen in liquid nitrogen and stored at − 80 °C until further analysis.

### DNA extraction

DNA extraction from digesta and mucosa samples was performed according to a previously described methodology^87^ with some modifications. Briefly, approximately 250 mg of digesta or mucosa samples were resuspended in 500 µl STE buffer (0.1 M NaCl, 10 mM Tris, 1 mM EDTA, pH 8) containing 0.4 g of glass beads (Sigma-Aldrich, G8772) and homogenized for 1 min at 6000 rpm on a Precellys 24 homogenizer (Bertin Instruments). Following 15 min incubation at 75 °C, with gentle agitation every 5 min, glass beads were removed by centrifugation and DNA extraction continued by incubating for 1 h at 37 °C, in the presence of 50 mg ml^−1^ lysozyme and 10 mg ml^−1^ RNAse, followed by a 30 min incubation at 55 °C with 20 mg ml^−1^ Proteinase K and 10% SDS. After 10 min on ice, in the presence of 500 µl of GES^87^ and 250 µl of ammonium acetate (7.5 M), a phenol-chloroform extraction was performed by adding 500 µl phenol-chloroform-isoamyl alcohol (25:24:1). The aqueous phase was re-extracted with 500 µl of chloroform-isoamyl alcohol (24:1) and the DNA of the subsequent aqueous phase was precipitated with 0.6 vol of isopropanol. After 10 min centrifugation at 13,000*g*, the DNA pellet was washed with ice-cold 80% ethanol and dried at room temperature. DNA was resuspended in 50 µl ultrapure water and stored at 4 °C.

### 16S rRNA genes sequencing and analysis

The taxonomic diversity of the European seabass allochthonous (digesta) and autochthonous (mucosa) gut microbiota concerning each feeding condition was comprehensively assessed by next-generation sequencing (NGS) technology. A total of 16 samples [8 digesta + 8 mucosa, being each sample a pool of 3 fish/tank] were sequenced using the Illumina MiSeq platform (Macrogen Inc., Seoul, Rep. of Korea), targeting the V3–V4 hypervariable region of the 16S rRNA gene, to obtain a sequence informative length of 300 bp. The paired-end (PE) reads were merged to produce longer reads using the Flash program^88^. Pre-processing (e.g. removal of low-quality reads) and Clustering was done using the CD-HIT-OTU program^89^. Initially, the filtered sequences were clustered at 100% identity into operational taxonomic units (OTU) identifying chimeric reads, and after removal of noise sequences (small size) the remaining representative reads from non-chimeric clusters were clustered into OTU at a 97% ID to species level cut-off. Singletons and low abundant (< 8) OTUs were removed from the analysis. Taxonomy assignment and diversity statistics were done using the Quantitative Insights Into Microbial Ecology (QIIME) software^90^ and SILVA^91^ 16S reference database.

### Statistical analysis

Data are presented as mean ± standard deviation. Statistical analysis was done by one-way ANOVA (growth performance, feed efficiency, and NGS data with Storey FDR correction for multiple testing) using the SPSS 21 software package for Windows (IBM SPSS Statistics, New York, USA) and STAMP v2.1.3 software^92^ for metagenomic profiles analysis. Data were tested for normality and homogeneity of variances by the Shapiro–Wilk and Levene’s test, respectively. When normality was not verified, data were transformed prior to ANOVA. Significant differences among groups were determined by the Tukey’s multiple range test. The probability level of 0.05 was used for rejection of the null hypothesis.

## Supplementary Information

Supplementary Tables.

## Data Availability

Raw sequences for this study can be found at NCBI Sequence Read Archive database (SRA; https://www.ncbi.nlm.nih.gov/sra) under the Bioproject accession number PRJNA606810.
